# Visualizable and Lubricating Hydrogel Microspheres Via NanoPOSS for Cartilage Regeneration

**DOI:** 10.1002/advs.202207438

**Published:** 2023-03-27

**Authors:** Yubin Yao, Gang Wei, Lianfu Deng, Wenguo Cui

**Affiliations:** ^1^ Department of Orthopaedics Shanghai Key Laboratory for Prevention and Treatment of Bone and Joint Diseases Shanghai Institute of Traumatology and Orthopaedics Ruijin Hospital Shanghai Jiao Tong University School of Medicine 197 Ruijin 2nd Road Shanghai 200025 P. R. China; ^2^ Department of Orthopaedics The First Affiliated Hospital of Wenzhou Medical University Wenzhou 325006 P. R. China

**Keywords:** cartilage repair, osteoarthritis, polyhedral oligomeric silsesquioxane (POSS), regeneration

## Abstract

The monitoring of tissue regeneration is particularly important. However, most materials do not allow direct observation of the regeneration process in the cartilage layer. Here, using sulfhydryl polyhedral oligomeric silsesquioxane (POSS‐SH) as a nano‐construction platform, poly(ethylene glycol) (PEG), Kartogenin (KGN), hydrogenated soya phosphatidylcholine (HSPC), and fluorescein are linked through the “click chemistry” method to construct nanomaterial with fluorescence visualization for cartilage repair: POSS linked with PEG, KGN, HSPC, and fluorescein (PPKHF). PPKHF nanoparticles are encapsulated with hyaluronic acid methacryloyl to prepare PPKHF‐loaded microfluidic hyaluronic acid methacrylate spheres (MHS@PPKHF) for in situ injection into the joint cavity using microfluidic technology. MHS@PPKHF forms a buffer lubricant layer in the joint space to reduce friction between articular cartilages, while releasing encapsulated positively charged PPKHF to the deep cartilage through electromagnetic force, facilitating visualization of the location of the drug via fluorescence. Moreover, PPKHF facilitates differentiation of bone marrow mesenchymal stem cells into chondrocytes, which are located in the subchondral bone. In animal experiment, the material accelerates cartilage regeneration while allowing monitoring of cartilage layer repair progression via fluorescence signals. Thus, these POSS‐based micro–nano hydrogel microspheres can be used for cartilage regeneration and monitoring and potentially for clinical osteoarthritis therapy.

## Graphical abstract

Based on the concept of “treatment‐monitoring integration”, a micro‐nano double lubricating hydrogel microsphere is fabricated through “click chemistry” method to construct POSS‐based nanomaterial in hydrogel microsphere combining poly(ethylene glycol), Kartogenin, hydrogenated soya phosphatidylcholine and fluorescein, which shows functional in situ cartilage regeneration and visualization, realizing the monitoring of tissue regeneration process.

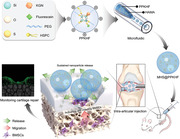

## 1 Introduction

Tissue regeneration is primarily used for the replacement, regeneration, and reconstruction of organs.^[^ ^1^ ^]^ The development of a new method to promote tissue regeneration requires better monitoring of the interaction between the engineered and host tissues, as well as the degree of tissue anastomosis. Currently, histopathological analysis is the principal method used to evaluate interactions between engineered and host tissues.^[^ ^2^ ^]^ Despite providing critical tissue information, this method does not allow monitoring of the tissue regeneration process. Moreover, the properties of biomaterials may be altered during histological analysis, which may lead to misleading results. With the advantages of long‐term monitoring of the morphological, functional, and molecular properties of tissues, biomedical imaging technology is widely used for monitoring tissue regeneration.^[^ ^3^ ^]^ However, there are very limited clinical technology options that can achieve low‐cost, zero‐radiation, and monitoring of the regeneration of damaged tissues. Thus, monitoring injured tissue regeneration remains a huge challenge for tissue engineering. Personalized medical technologies that can monitor and track treatment outcomes and disease conditions are critical for the success of precise treatment.

Currently, tissue regeneration is mainly monitored by X‐ray imaging, computed tomography (CT) imaging, magnetic resonance imaging (MRI), radionuclide imaging, and in vivo optical imaging.^[^ ^4^ ^]^ X‐ray and CT imaging present differences in the density of tissue structures through grayscale images generated by multilayer scans of the human body; however, both have poor visualization capability and low resolution for cartilage tissue. MRI has better resolution than X‐ray and CT for soft tissue and is radiation‐free. However, its application in routine monitoring of cartilage regeneration is restricted by its slow imaging speed. In addition, these imaging methods generally require additional contrast agents and cannot acquire high‐definition images from samples with metallic materials in vivo. In recent years, in vivo optical imaging has developed rapidly; in vivo fluorescence imaging is especially favored by researchers for its high specificity and sensitivity, high spatial resolution, clean background, quantitative analysis, low tissue damage, clinical safety, and wide application.^[^ ^5^ ^]^ Generally, there are two ways to promote tissue regeneration: the immune system promotes tissue regeneration around the injury site, and tissue‐engineered biomaterials carry and release drugs capable of stimulating tissue regeneration. Biomaterials are widely used in tissue repair because of their injectability, degradability, high biosafety, and stable drug delivery;^[^ ^6^ ^]^ however, most of them have only a single function of promoting tissue repair let alone monitoring the process during tissue regeneration. Thus, it is particularly important to design and develop biomaterials that promote tissue repair, while allowing monitoring of the process.

Osteoarthritis (OA) is a degenerative disease characterized by dysfunction of joint lubrication and wear and tear of the joint cartilage, resulting in joint pain, limitation of motion, and loss of function, which seriously affects the quality of life of patients.^[^ ^7^ ^]^ OA is usually treated with conservative and surgical methods, while intra‐articular injection of drugs is the preferred conservative treatment method.^[^ ^8^ ^]^ In recent years, a variety of intra‐articular injections has emerged in the preclinical research stage, such as the development of RNA or DNA delivery systems based on in vivo nucleic acid transfection.^[^ ^9^ ^]^ Mesenchymal stem cells (MSCs) have become popular injectable materials for repairing cartilage injury through intra‐articular injection.^[^ ^10^ ^]^ Although the above‐mentioned therapeutics promoted healing in OA joints, the repair was not monitored and may lead to insufficient or excessive cartilage regeneration. Insufficient cartilage repair fails to fully recover the cartilage surface, which increases the possibility of OA recurrence after long‐term mechanical friction between the upper and lower articular surfaces. Hence, it is imperative to develop biomaterials that can simultaneously promote cartilage repair and monitor the cartilage repair process to accurately cure the disease.

Polyhedral oligomeric silsesquioxane (POSS), a class of organic/inorganic nanohybrid materials with a special cage‐like structure, has the chemical formula (RSiOl.5) *n*, where *n* is an even integer greater than or equal to 6, and the R group may be an organic or inorganic group, which may also be an atom or a group with strong reactivity.^[^ ^11^ ^]^ POSS is widely used in biomaterials because of its unique Si—O—Si framework and the nature of nano‐hard materials that can be easily taken up by cells.^[^ ^12^ ^]^ Han et al. introduced OV‐POSS into hydrogels, forming well‐dispersed nanocomposites with precisely size‐controlled hard blocks within it and increasing the crosslinking density to improve mechanical properties for repairing osteochondral defects.^[^ ^13^ ^]^ From drug delivery to bioimaging, the distinctive advantages of POSS: easy functionalization, chemical stability, and biocompatibility have allowed researchers to develop better drug delivery systems and contrast imaging platforms.^[^ ^14^ ^]^ Kartogenin (KGN), as a mature small‐molecule drug, its pharmacological mechanism of promoting chondrogenic differentiation of stem cells by activating PI3‐Akt pathway through its cleavage product 4‐ABP has been confirmed by Shuai Zhang et al. and has been widely used in medical research.^[^ ^15^ ^]^ Compared to the traditional cytokines, TGF‐*β* for example, KGN has many advantages, such as stable drug activity and low price. Moreover, as a result of the hydration lubrication property of the phospholipid head base of hydrogenated soya phosphatidylcholine (HSPC), there will form a super‐lubricating film on the cartilage surface, which can effectively reduce the friction coefficient of the lesion area in the process of promoting the regeneration of injured cartilage so as to protect cells from secondary friction damage and create good regeneration conditions for the damaged tissue. HSPC has a high phase transition temperature (50 °C), which enables good lubrication performance both at room temperature and in vivo (37 °C).^[^ ^16^ ^]^

**Scheme 1:**
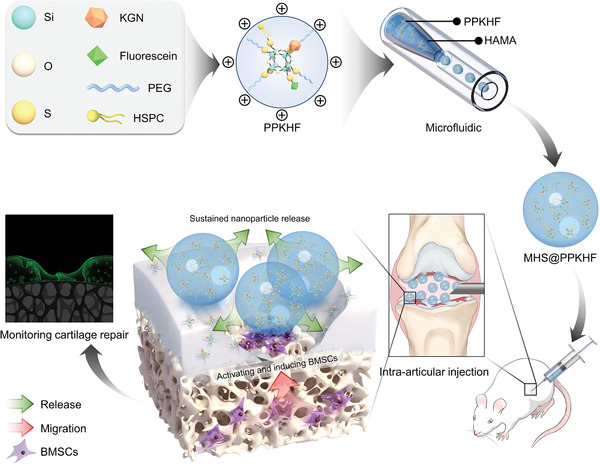
Based on the concept of “ treatment‐monitoring integration,” a micro–nano double lubricating hydrogel microsphere with functions of in situ cartilage regeneration and repair process visualization was designed, which realized the monitoring of tissue regeneration process. Nanoparticles carrying KGN penetrated the cartilage layer and promoted the proliferation and chondrogenic differentiation of bone marrow mesenchymal stem cells (BMSCs) distributed in subchondral bone. At the same time, the regeneration process of cartilage layer was visualized by fluorescein (MHS@PPKHF: PPKHF‐loaded microfluidic hyaluronic acid methacrylate spheres; PPKHF: POSS linked with PEG, KGN, HSPC, and fluorescein).

In this study, using sulfhydryl polyhedral oligomeric silsesquioxane (POSS‐SH) as a nano‐construction platform, polyethylene glycol (PEG), Kartogenin (KGN), hydrogenated soya phosphatidylcholine (HSPC), and fluorescein were chemically grafted together using one‐step “click chemistry” to form a stable and positively charged multifunctional POSS hybrid molecule: POSS linked with PEG, KGN, HSPC and fluorescein (PPKHF) that combines fluorescence imaging, promotion of cartilage repair, and enhanced joint lubrication.^[^ ^15^ ^]^ PPKHF was then uniformly mixed with methacrylated hyaluronic acid to prepare lubricated hydrogel microspheres, that is, PPKHF‐loaded microfluidic hyaluronic acid methacrylate spheres (MHS@PPKHF) using a unique microfluidic technology,^[^ ^17^ ^]^ which can gradually degrade and slowly release PPKHF for the treatment and monitoring of OA (**Scheme** **1**). The relationship between material structure and properties was revealed by spectral characterization and structural analysis. The biological effects of MHS@PPKHF on rat bone marrow mesenchymal stem cells (BMSCs) were investigated in detail, and the monitoring effect of this material during regeneration in the rat OA model was further evaluated using in vivo fluorescence imaging technology and pathological sections.

## 2 Results and Discussion

### 2.1 Preparation and Characterization of Visualized Nanoparticles

**Figure 1:**
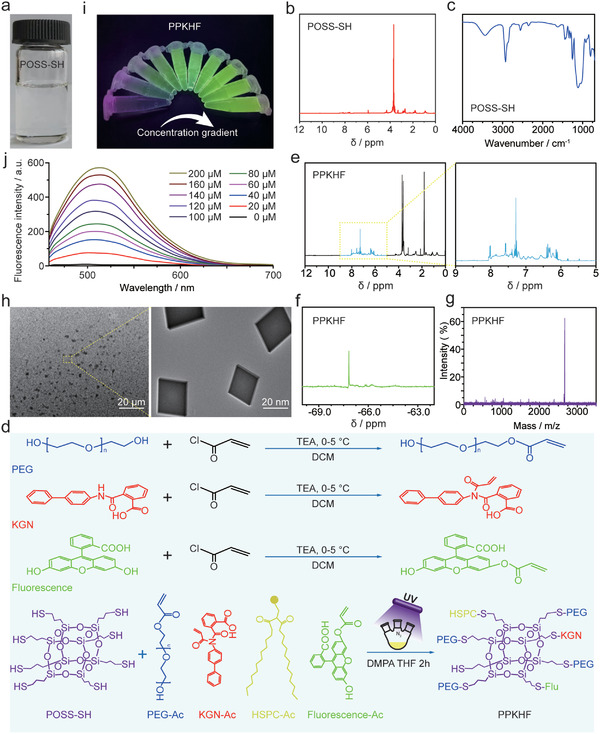
a) Photo of finished POSS‐SH product; b) nuclear magnetic hydrogen (NMH) spectrum of POSS‐SH; c) Fourier transform infrared (FTIR) spectroscopy of POSS‐SH; d) synthesis process of PPKHF; e) NMH spectrum of PPKHF; f) silicon spectrum of PPKHF; g) mass spectrum of PPKHF; h) TEM images of PPKHF; i) fluorescence photos of PPKHF at series of concentrations under 365 nm UV lamp; and j) fluorescence intensity of different concentrations of PPKHF.

To improve the visualization of the therapeutic effect of OA, POSS‐based micro–nano lubricating hydrogel microspheres were designed and synthesized. Then, using POSS as a nano‐construction platform, POSS‐SH was prepared by hydrolytic condensation in the form of a colorless transparent viscous liquid (**Figure** **1**a), with the structure shown to be pure by nuclear magnetic hydrogen (NMH) spectroscopy (Figure 1b) and FTIR spectroscopy (Figure 1c). Furthermore, KGN, fluorescein, PEG, and phospholipid acetylcholine were chemically linked together using “click chemistry” to prepare PPKHF (Figure 1d), whose decoupling was characterized by NMH spectroscopy, NMR silica spectroscopy, and mass spectrometry. At 1.5 ppm, it exhibited an unreacted thiol peak, a characteristic peak of a benzene ring between 6 and 8 ppm (Figure 1e), and according to the silicon spectrum (Figure 1f), there was only one characteristic peak of the silicon spectrum at _−_67.5 ppm with a molecular weight of 277.54 (Figure 1g), indicating that PPKHF was successfully synthesized with an average particle size of 27 nm (Figure 1h). After modification by POSS‐SH, KGN molecule spacing increased, which made it difficult to aggregate. Meanwhile, the introduction of PEG greatly increased the overall water solubility of PPKHF, which made the modified KGN have good water solubility. PPKHF exhibited good fluorescence visualization under 365‐nm UV lamp irradiation (Figure 1i), and quantitative fluorescence spectroscopy demonstrated that the fluorescence intensity increased linearly with increasing concentrations from 0–200 µm (Figure 1j).

### 2.2 Preparation, Characterization, and Lubrication Properties of Visualized MHS@PPKHF

**Figure 2:**
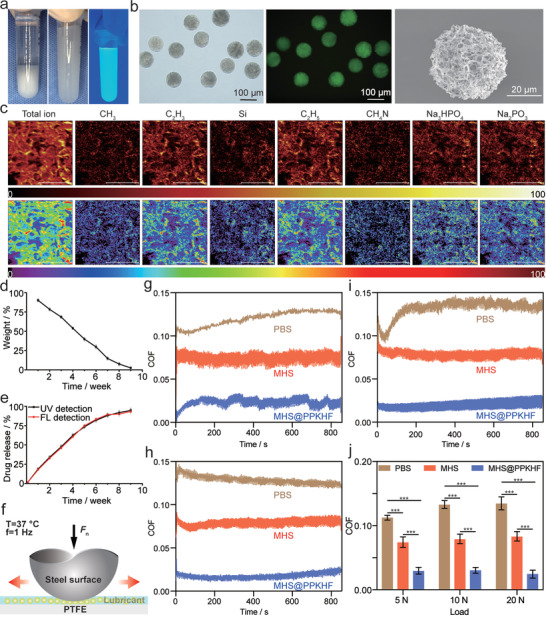
a) State of MHS@PPKHF after standing and precipitating, shaken into suspension and under UV light irradiation; b) hydrogel microspheres under bright field microscope, fluorescence microscope, and electron microscope; c) flight secondary ion mass spectroscopy(F‐SIMS) results of MHS@PPKHF; d) degradation curve of MHS@PPKHF; e) drug release profile; f) friction test diagram; and g–j) friction curves and statistical graphs. (* *p* < 0.05, ** *p* < 0.01, and *** *p* < 0.001).

To enable PPKHF to slowly visualize the effect of the drug in the OA model, highly monodispersed hydrogel microspheres (MHS@PPKHF) were prepared by homogeneously mixing PPKHF with HA as the raw material using microfluidic technology. MHS@PPKHF could settle after standing in an aqueous solution and forming a translucent, uniformly dispersed suspension after it was shaken gently, which is visible as a uniform green fluorescence under UV light and can maintain structural stability for ≈30 min at room temperature (**Figure** **2**a). The MHS@PPKHF had an overall particle size distribution of ≈100 µm and appeared as green fluorescence under fluorescence microscopy (Figure 2b), which guarantees its visualization in the OA model. MHS@PPKHF was ≈40 µm in size after lyophilization, with a porous structure as a whole. An F‐SIMS analysis was performed (Figure 2c; Figure S1, Supporting Information) to further validate the successful preparation of MHS@PPKHF. It should be noted that the multifunctional hydrogel microspheres were fabricated homogeneously, and the Si elements inside the POSS structure were uniformly distributed inside MHS@PPKHF. In addition, it was observed that the P element of phospholipid acetylcholine, the benzene ring of KGN, the hydroxyl group of PEG, and the characteristic functional group of fluorescein were all distributed around the Si element, and all of these elements and groups were uniformly dispersed in MHS@PPKHF. The above results indicate the successful synthesis of PPKHF, and evenly dispersed hydrogel microspheres can be prepared with hyaluronic acid gel, which provides a favorable material basis for subsequent visual therapy.

The ability to maintain a long retention time and stable drug release in the articular cavity is a key indicator of intra‐articular injections for OA treatment. Thus, the degradation properties and drug‐release characteristics of the MHS@PPKHF were investigated. Degradation experiments were performed in PBS solution containing 0.2% type II collagenase. The hydrogel microspheres were weighed and observed for morphological changes under a microscope at fixed time points each week. As shown in Figure 2d, the MHS@PPKHF degradation was a slow and relatively stable process. As shown in the microscopic observation image (Figure S3, Supporting Information), the overall structure of the microspheres was relatively intact, and the particle size did not change significantly in the first 2 weeks. Thereafter, MHS@PPKHF gradually lost its structural integrity as the surface became coarse and cracked, with a continuous decrease in the particle size and number, and almost completely degraded at the 9th week. The process of sustained release of PPKHF from the microspheres was also measured in PBS at 37 °C, and the release curves were plotted according to the changes in UV absorbance and fluorescence intensity. The change in drug concentration measured using the two methods presented similar trends, and the overall trend was in two phases (Figure 2e). PPKHF was released quickly in the first 5 weeks, and the curve slope was large, indicating the outbreak phase of drug release. Subsequently, the curve tended to flatten, and the drug release gradually decreased, entering a relatively stable phase. This result demonstrated that MHS@PPKHF had a persistent drug release behavior, which would be beneficial for long‐term administration at the focal point after injection.

Moreover, a super‐lubricating film on the cartilage surface was formed by utilizing the hydration lubrication property of the HSPC phospholipid head base, which can improve the lubricating property of the articular cartilage surface and reduce the damage to cartilage caused by mechanical friction.^[^ ^16^ ^]^ A schematic diagram of the friction tests in this study is shown in Figure 2f, and the friction pair was composed of polished stainless‐steel balls and PTFE plates. The frequency of knee joint activity during normal walking is ≈0.5 Hz, while it can reach 2–3 Hz during running.^[^ ^18^ ^]^ Therefore, 1 Hz was selected as the sliding frequency of the friction pair to detect the lubricating performance of the MHS and MHS@PPKHF in a human body temperature environment. Representative friction coefficient‐time curves are plotted under different loads (Figure 2g–i). Given that the friction process was performed in a PBS solution, the PBS group was used as a blank control for each group of tests. Statistical analysis of the coefficients of friction (COF) under three different loads of 5, 10, and 20 N (Figure 2j) suggested that the COFs of the PBS group were the largest under different loads, followed by the MHS group, and the MHS@PPKHF group was the smallest. As the load increased, the COFs of both PBS and MHS groups presented a certain upward trend, while the COFs of the MHS@PPKHF group were stable between 0.02 and 0.03 (at least five times higher than the PBS group in the lubrication performance), suggesting that as the outer hyaluronic acid gel was worn off, the PPKHF encapsulated inside the microspheres continuously replenished the lubrication layer to ensure long‐term stable lubrication performance of the material. It is worth noting that, compared with the loads of 5 and 10 N, the COF of the MHS@PPKHF group under a high load of 20 N decreased to 0.024 instead of increasing, which fully meets the requirement of joint lubrication (<0.03).^[^ ^19^ ^]^ These results fully demonstrate the excellent hydrated lubricating property of MHS@PPKHF, which enables it to provide high‐efficiency double lubrication for articular cartilage in a physiological environment.

### 2.3 Evaluation of Biocompatibility and Chondrogenic Induction of Visualized MHS@PPKHF

**Figure 3:**
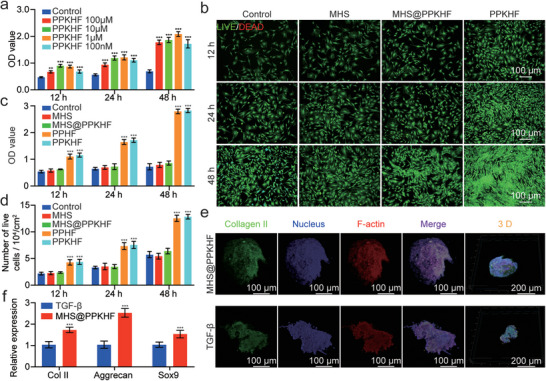
a) Cell proliferation under different concentrations of PPKHF (*n* = 3, * *p* < 0.05, ** *p* < 0.01, and ***p < 0.001); b) representative fluorescence images of MHS, MHS@PPKHF, and PPKHF co‐cultured BMSCs by live‐dead staining assay; c) CCK8 values measured with live‐dead staining (*n* = 3, * *p* < 0.05, ** *p* < 0.01, and *** *p* < 0.001); d) viable cell count results (*n* = 3, * *p* < 0.05, ** *p* < 0.01, and *** p < 0.001); e) fluorescence confocal microscopic images of cell clusters induced by MHS@PPKHF and TGF‐*β*; f) mRNA relative expression levels of Collagen II, Aggrecan, and Sox9 (*n* = 3, * *p* < 0.05, ** *p* < 0.01, and *** *p* < 0.001, compared with TGF‐*β* group).

To investigate the cytotoxicity and chondrogenic induction of multifunctional MHS@PPKHF and the loaded PPKHF hybrid nanoparticles on BMSCs in vitro, PPKHF in culture medium was prepared in a series of concentration gradients (100 µm, 10 µm, 1 µm, and 100 nm) for the culture of BMSCs. Meanwhile, basal medium without PPKHF was used as the control group, and the CCK‐8 assay was performed on the cells after 12, 24, and 48 h of culture to determine the optimal drug concentration for cell growth. As shown in **Figure** **3**a, different concentrations of PPKHF at all time points showed a significant positive promotion of cell growth and proliferation compared with the control group. Therefore, 1 µm was used as the optimal concentration of PPKHF for subsequent experiments. Unloaded hydrogel microspheres MHS, MHS@PPKHF (hybrid hydrogel microspheres), 1 µm PPHF, and 1 µm PPKHF were co‐cultured with BMSCs and then subjected to live–dead staining (Figure 3b; Figure S4, Supporting Information), CCK‐8 detection (Figure 3c), and cell count analysis (Figure 3d) after 12, 24, and 48 h, respectively. The cell density increased with culture time in all four groups, and almost all cells in each group were viable, with few dead cells observed. Compared with the control group, there was no significant difference in cell proliferation activity between the MHS and MHS@PPKHF groups at any time point, whereas the cell proliferation activity of the PPHF and PPKHF group far exceeded that of the previous three groups, exhibiting a striking pro‐proliferative ability. This result is attributed to the POSS skeleton, which is beneficial to cell adhesion and detachment while improving the mechanical rigidity of the cell structure.^[^ ^12^ ^]^ The failure of MHS@PPKHF group to significantly promote cell proliferation may be because the microsphere co‐culture time with cells is too short to degrade and release effective drug concentration during this period. In general, the above results indicate that the materials used in this study have good biocompatibility with BMSCs and can effectively promote cell proliferation.

In addition, the chondrogenic induction ability of MHS@PPKHF was evaluated. As previously reported, the appropriate concentration of KGN for chondrogenic differentiation of stem cells in vitro was between 1 and 10 µm, and long‐term stable maintenance of this concentration will be beneficial to this process. As a growth factor essential for the induction of chondrogenic differentiation of stem cells and an important component of the in vitro stem cell chondrogenic induction medium, TGF‐*β* is widely used in various cartilage regeneration engineering scaffolds. However, strict preservation conditions and the high market prices of growth factors have hindered their widespread clinical application. Our study aimed to replace TGF‐*β* with KGN, a more economical and stable small‐molecule drug, for chondrogenic induction. The leachate of MHS@PPKHF was used for the induction culture of BMSCs, whereas TGF‐*β* was used as the control group. Last, immunofluorescence staining was performed on the induction results to determine the expression level of type II collagen in cell masses (Figure 3e). Moreover, the expression levels of three chondrocyte markers (Col2a1, Aggrecan, and Sox9) were compared between the two groups (Figure 3f). The expression levels of three chondrocyte markers in the MHS@PPKHF group were significantly higher than those in the TGF‐*β* group.

### 2.4 Visualized Monitoring of Drug Retention in Articular Cavity and Cartilage Repair Process

**Figure 4:**
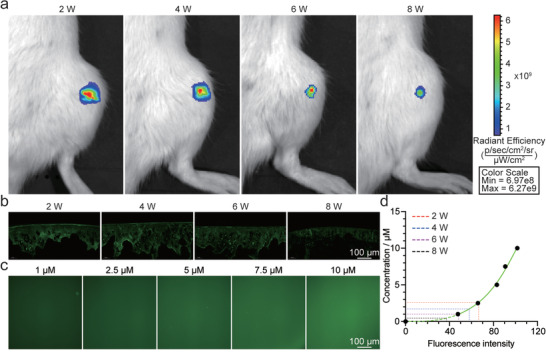
a) In vivo imaging system (IVIS) images of visualized MHS@PPKHF in the articular cavity of rat at different time points; b) images of rat knee slices under fluorescence microscope; c) fluorescence images of PPKHF at different concentrations; and d) graph of fluorescence intensity–drug concentration.

Long‐term stable release of the drug in the joint cavity is a key factor in improving the efficiency of cartilage regeneration in vivo. To explore the retention effect of MHS@PPKHF in the articular cavity, fluorescent molecules were grafted onto PPKHF nanoparticles and the changes of fluorescence signal in the joint cavity after operation by an in vivo imaging system (IVIS) (**Figure** **4**a) were observed. After the intra‐articular injection of MHS@PPKHF at the second week after surgery, the fluorescence signal in the joint cavity remained at a relatively stable level for most of the time in the future, and the fluorescence intensity gradually decreased with the gradual degradation of MHS@PPKHF. Until the eighth week, a weak fluorescence signal was still visible in the joint cavity, indicating that MHS@PPKHF had excellent drug release performance and retention effect in the articular cavity. It has been proven that positively charged nanocarriers have a strong ability to penetrate negatively charged cartilage tissue; thus, breaking through the biological barrier to achieve efficient drug delivery.^[^ ^20^ ^]^ The surface charge of PPKHF was detected, and the results indicated that the nanoparticles exhibited cartilage penetration with an average zeta potential of 34.2 mV (Figure S6, Supporting Information). To further verify this conclusion, cryosections of the MHS@PPKHF group were imaged under a fluorescence microscope at the 2nd, 4th, 6th, and 8th week of treatment. Part of the meniscus remained above the tibial plateau, which would interfere with the penetration of PPKHF into the cartilage of the tibial plateau region. Therefore, for the sake of experimental rigor, we selected cartilage tissue from the femoral medial condyle region for observation. The results showed (Figure 4b) that PPKHF was evenly distributed in the cartilage layer, even as deep as the subchondral bone, and that the fluorescence intensity remained stable for a long period. It can be observed that the material has excellent penetration of cartilage tissue. In order to further explore the actual concentration of PPKHF in cartilage tissue, fluorescence images of different concentrations of PPKHF were obtained under the same shooting parameters (Figure 4c). Image J software was used to make statistics on fluorescence intensity and a graph of fluorescence intensity to drug concentration was drawn according to the statistical results (Figure 4d). At the same time, the fluorescence intensity of the sections was quantified and the PPKHF concentration in the cartilage tissue of the 2nd, 4th, 6^th^, and 8th week turned out to be 2.4, 1.7, 0.9, and 0.4 µm, respectively. From the above results, the actual concentration of PPKHF infiltrating into cartilage tissue was between 1 and 10 µm during most of the treatment period, which was also in line with the optimal concentration range obtained in cell experiments.

### 2.5 Evaluation of In Vivo Treatment of OA

**Figure 5:**
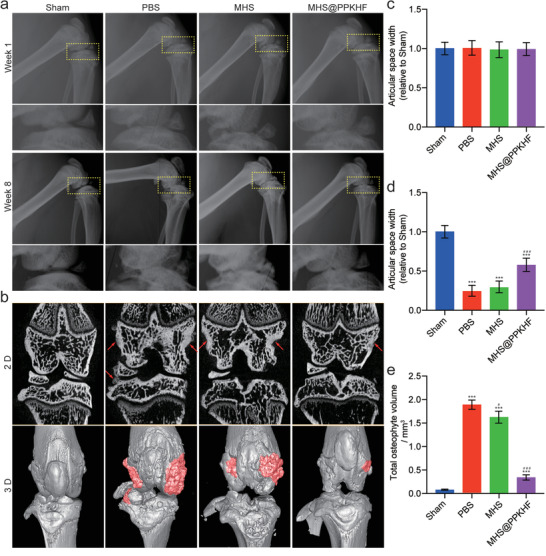
a) Lateral X‐ray films of rat knees at 1st and 8th week after destabilization of medial meniscus (MMx), b) micro‐CT tomographic images and 3D reconstruction images of rat knee joints at 8 weeks postoperation, c) relative joint gap width of each group at 1‐week postoperation, d) relative joint gap width of each group at 8 weeks postoperation, and e) total volume of knee osteophytes in each group at 8 weeks postoperation. (*n* = 3, *** and ### indicate *p* < 0.001 when compared with Sham group and PBS group, respectively).

The OA model constructed based on partial medial meniscectomy (MM*x*) is more consistent with the pathogenesis of chronic OA than OA models based on other induction procedures (e.g., anterior cruciate ligament transection). Intra‐articular injection of MHS@PPKHF was performed 2, 5, and 8 weeks postoperatively. After 8 weeks, all rats were euthanized and their knees were evaluated for efficacy. X‐ray imaging of the knees was performed at the first and eighth week after surgery (**Figure** **5**a). From the images, there was no significant difference in the width of the joint space between the groups in the first week (Figure 5c) but a significant narrowing of the joint space was observed at week 8 in the PBS and MHS groups. Although the MHS@PPKHF group had a narrower joint gap width than the sham‐operation group, it still presented a good therapeutic effect compared to the other two groups (Figure 5d). X‐ray images provide very limited information for the diagnosis and assessment of OA efficacy. Hence, a more comprehensive assessment of the knee joint samples of each group was performed using Micro‐CT at 8 weeks after surgery (Figure 5b).^[^ ^21^ ^]^ The subchondral bone of the knee was significantly thickened, and different degrees of subchondral osteosclerosis occurred in the PBS and MHS groups compared to that in the sham‐operation group, but this was not evident in the MHS@PPKHF group (Figure S7, Supporting Information). In addition, as shown in Figure 5e, the osteophyte volumes of the samples from each group were measured using a computer. The PBS group with a total osteophyte volume (TOV) of 1.92 ± 0.09 mm^3^ was the worst, while the MHS group with a TOV of 1.61 ± 0.12 mm^3^ was not good either. There was no significant difference in total osteophyte volume between MHS @ PPKHF group (0.22 ± 0.10 mm^3^) and the sham operation group (0.13 ± 0.07 mm^3^), demonstrating the excellent anti‐OA performance of the material.

**Figure 6:**
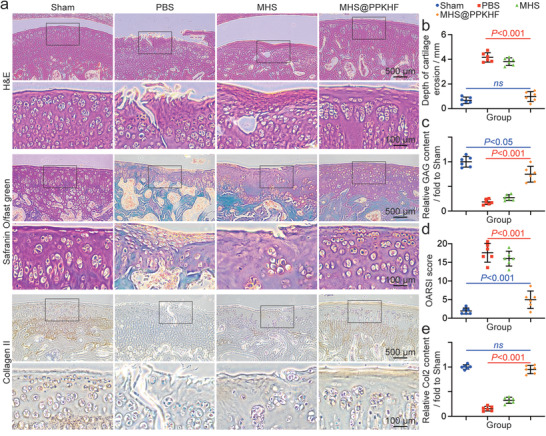
a) H&E staining, Safranin O‐fast green staining, and type II collagen immunohistochemical staining of each group; b) cartilage wear depth; c) relative glycosaminoglycan (GAG) content; d) OARSI score of each group, and e) relative type II collagen content. (*n* = 3, ns: no significance).

Histological analysis was performed on tissue sections from the knee joints of each group to further evaluate the anti‐OA performance of MHS@PPKHF. According to the histological evaluation of cartilage based on hematoxylin‐eosin (H&E) staining and Safranin O‐fast green staining (**Figure** **6**a), typical pathological manifestations of osteoarthritis were observed in the PBS group, such as coarse cartilage surface, cartilage tissue erosion and deformation, and longitudinal cracks of the cartilage layer. The gross erosion depth of cartilage layer in PBS group was 4.08 ± 0.13 mm (Figure 6b). Compared to the PBS group, the performance of the MHS group was greatly improved but it was still not optimistic. The columnar structure of cartilage tissue and the integrity of surface cartilage were best maintained in the MHS@PPKHF group, without obvious tissue exfoliation and deformation, and tissue cell cloning increased to a certain extent. There was no significant difference in cartilage erosion depth compared to the sham operation group (negative control). According to the results of Safranin O‐fast green staining, compared with the PBS group and MHS group, the relative content of glycosaminoglycan (GAG) in the MHS@PPKHF group was the highest (Figure 6c), showing a better performance in maintaining cartilage matrix. Scores by the Osteoarthritis Research Society International (OARSI) assessment protocol (Figure 6d) revealed that, except for the MHS@PPKHF group, the scores of the other two groups showed a significant increase 8 weeks after MM*x*, which further verifies the excellent anti‐OA performance of MHS@PPKHF. Furthermore, the sections of the knee joint from each group were subjected to immunohistochemical staining for type II collagen (the main biomarker of cartilage), and the relative content in each group was measured (Figure 6e). Compared with the sham operation group, the relative content of type II collagen was significantly reduced to ≈17% and 35% in the PBS and MHS groups, respectively. However, the average type II collagen content in samples from the MHS@PPKHF group was ≈93% of that in the negative control group, without a significant difference between the two groups. There were still differences in GAG content and OARSI score between the MHS@PPKHF group and the sham group (Figure 6c,d), which could not completely restore OA cartilage to the perfect state before injury, but these gaps had been greatly reduced comparing to the results of PBS group and MHS group. In general, the above results indicate that MHS@PPKHF has a more prominent performance in promoting cartilage tissue regeneration. Therefore, this is a desirable method for the treatment of OA.

## 3 Conclusion

In this study, with POSS‐SH as the nano‐construction platform, multifunctional nanoparticles integrating lubrication, cartilage regeneration, and visualization via fluorescence imaging were successfully synthesized by the “click chemistry” method. Highly monodisperse multifunctional hydrogel microspheres in uniform size were fabricated through microfluidic technology. The material exhibited excellent hydration lubrication properties with a sufficiently low COF value (<0.03) under a high load, which fully meets the requirements for joint lubrication in physiological environments. Moreover, MHS@PPKHF exhibited an unexpected ability to promote cell proliferation and induce chondrogenic differentiation of BMSCs. In the rat OA model, MHS@PPKHF had a strong retention effect in the articular cavity, facilitating monitoring of drug metabolism through visualization, which would be conducive to personalized treatment and observation in clinical practice. In addition, PPKHF possesses a strong cartilage penetration ability owing to its positive charge, which greatly improves the drug delivery effectiveness and further understanding the healing of cartilage tissue through visualization technology. Compared with traditional pathological sections, the fluorescence visualization monitoring technology based on MHS@PPKHF skips the tedious staining operation, provides a more convenient and quicker path for observing the progress of cartilage repair, but also better guarantees the effectiveness of observation results. However, most of our therapeutic effects in monitoring of cartilage tissue were based on in vitro results, not in vivo. Our current research mainly realizes the monitoring of tissue regeneration in vitro and has potential tracking effect on the repair process of cartilage tissue in vivo. Markers, such as collagen X and pro‐inflammatory mediators (such as IL‐1 and TNF), are also important indicators for the evaluation of OA prognosis. However, as this study focused on the promotion and monitoring of cartilage tissue regeneration, there was no further discussion on the anti‐inflammatory properties of materials, which is one of the limitations of this study. It is still possible to reverse cartilage damage in the early stage of osteoarthritis, which is the golden period for treatment. The purpose of this study was to seize the early stage of the disease to implement salvage repair of cartilage damage; so, a short observation period was selected in the animal experiment. However, there was no further investigation into the late course of disease, which is also a limitation of the study. With the current positive results, we plan to extend the period of animal experiments in subsequent studies; so as to conduct more in‐depth research on cartilage repair in the advanced stage of osteoarthritis. In conclusion, the visualized multifunctional MHS@PPKHF prepared in this study provides a strategy with clinical application potential for accurate and individualized treatment of OA cartilage repair. This visualization system can be extended to other clinical treatment areas (e.g., treatment of major organs of the body, such as the digestive, respiratory, and urinary systems).

## 4 Experimental Section

### Material Reagents

KGN (CAS: 4727‐31‐5) was purchased from MedChemExpress (USA) and HSPC (CAS: 97281‐48‐6) was purchased from Avanti Polar Lipids (USA). Reagents such as (3‐Mercaptopropyl)trimethoxysilane, concentrated hydrochloric acid (37%), acryloyl chloride, tetrahydrofuran, azobisisobutyronitrile, triethylamine, anhydrous ethanol, anhydrous dichloromethane, *N*,*N*‐dimethylformamide, dimethyl sulfoxide, anhydrous sodium sulfate, sodium chloride, sodium hydroxide, anhydrous magnesium sulfate, 4‐A molecular sieve, potassium bromide, deuterated chloroform, deuterated dimethyl sulfoxide, silica gel, benzophenone, polyethylene glycol (PEG), and potassium hydroxide phosphorus pentoxide were purchased from Sigma–Aldrich. POSS‐SH and fluorescein were synthesized in a previous work.^[^ ^22^ ^]^ All reagents were analytical grade that could be used without further purification.

### Fluorescence Spectroscopy

Fluorescence excitation spectra and emission spectra were measured using an LS55 fluorescence spectrometer from PE, and the solution to be tested was placed in a 1 cm quartz cuvette.

### UV–Visible Absorption Spectroscopy

The test was performed using a Lambda 35 UV absorption spectrometer from PE, with wavelength range of 800–200 nm and a step size of 1 nm.

### Synthesis of PPKHF

POSS‐SH was added to a 50.0 mL three‐necked flask and dissolved with (0.800 mol) of PEG‐Ac and 0.0600 g (0.0600 mmol) of Dimethylolpropionic Acid (DMPA) in 10.0 mL of tetrahydrofuran, followed by irradiation reaction at room temperature for 2.5 h under a 365 nm hand‐held UV lamp. After that, 0.0620 g (0.200 mmol) of KGN‐Ac, 0.1200 g (0.400 mmol) of DMPA, and hydrogenated soya phosphatidylcholine were dissolved in 5.00 mL of tetrahydrofuran, followed by irradiation reaction for 6 h under a UV lamp continuously. Last, the solvent was removed by rotary evaporation to obtain the target sensing material PPKHF and the yield was 90.2%. 1H NMR (CDCl3, 600 MHz, ppm): *δ* = 0.63 (d, Si—CH2—), 1.36 (—SH), 1.62 (s, —CH2—), 2.52 (s, _−_CH2_−_ S), 3.55 (s, CH2_−_OH), 4.14 (s, OH), 6.22 (s,), 6.32, 6.87–8.05 (d, ArH), 10.91–10.23 (d, _−_NH). 13C NMR (600 MHz, CDCl3‐d, 298K, *δ*/ppm): *δ* = 12.3, 16.5, 22.5, 31.1, 33.4, 39.6, 49.7, 60.4, 63.2, 66.3, 68.1, 70.4, 71.4, 97.8, 103.1, 120.3, 122.2, 124.5, 126.1, 127.5, 132.6, 133.7, 136.5, 165.2, 174.7, 194.6. 29Si NMR (600 MHz, CDCl3‐d, 298 K, *δ*/ppm): *δ* = _−_66.45 (s); MALDI‐TOF‐MASS m/z calcd. POSS‐Rha‐PEG‐SP: 3435.3, found: 3435.1.

PPKHF was dissolved in water (1 mm) and stirred at room temperature for 4 h after adding zinc chloride solution (0.01 mm). After the reaction, PPKHF with positive charge was obtained by dialysis for 2 days (molecular weight 2000).

### Synthesis of HAMA

Hyaluronic acid methacrylate (HAMA) was synthesized following the procedure previously described.^[^ ^23^ ^]^ In PBS at 60 °C, hyaluronic acid was dissolved completely to produce a 10% w/v homogenous solution. After that, methacrylic acid was added to react with HA (1200 kDa) for 1 h at 50 °C. The reaction was stopped by adding PBS diluent five times intermittently; and then, the impurities were filtered by dialyzing with a 14 kDa cutoff analyzer tube for 1 week at 40 °C. After lyophilization of HAMA aqueous solution, white porous foam was obtained.

### Synthesis of MHS@PPKHF

An improved microfluidic device was used to prepare MHS@PPKHF. To be brief, the aqueous phase (4 wt% HAMA, 1 wt% PPKHF, and 0.5 wt% light stabilizer mixed in PBS evenly) and the oil phase (paraffin oil containing 5 wt% span80) were injected into the microfluidic device. A syringe was attached to the pump, controlling the flow rate of two phases. In this study, the flow rates of oil phase and water phase were set to 2000 and 40 µL min^−1^ respectively. A UV cross‐link process was then applied to the obtained monodisperse emulsion drops. The resulting microspheres were collected in test tubes and washed with isopropyl alcohol by shaking, and then centrifuged at 4000 rpm for collection of microspheres.

### Drug Release Test In Vitro

PPKHF solutions with a range of concentration gradients were prepared with PBS (pH = 7.4) and the absorbance was recorded at 276 nm using a UV–vis spectrophotometer (UV‐6100S, Metash Instruments, China), and the corresponding fluorescence intensity was recorded simultaneously to obtain the calibration curve.

The tube with 5 mL of MHS@PPKHF suspension was placed on a thermostatic shaker (37 °C, 80 rpm). The tube was removed and centrifuged (4000 rpm, 10 min) to separate the phases, and 1 mL of the supernatant was used for detection of UV absorbance and fluorescence intensity. After detection, the solution was refilled into the test tube and continued to be shaken at a constant temperature until the microspheres were completely degraded.

### In Vitro Degradation Test

The degradation of HAMA hydrogels was carried out in PBS solution of collagenase mimicking physiological environment in vivo. At a word, 10 mg of MHS and 10 mg of MHS@PPKHF were, respectively dispersed in 5 mL of PBS at 37 °C and digested with type II collagenase at a concentration of 0.2%. Collagenase solution was supplemented every 2 days for enzyme activity maintaining. The degradation of MHS and MHS@PPKHF was analyzed by observing morphological changes and measuring residual weight at fixed time points every week.

The degradation rate (DR) was calculated by following formula:

(1) $$\mathrm{DR}\;\;\left(\%\right)=\;1-\frac{\mathrm{Weight}\;\mathrm{at}\;Wx}{\mathrm{Initial}\;\mathrm{weight}}\;\times 100\%$$

wherein, *Wx* refers to week *x*.

### Friction Test

The lubrication measurements of MHS@PPKHF aqueous suspension were carried out using an UMT‐3 Universal Testing Machine (Brooknami, Germany) under 5, 10, and 20 N loads. 4 mm and 1 Hz were set for reciprocating sliding distance amplitude and sliding frequency, respectively. The test lasted 15 min each time. The friction pair was composed of a polytetrafluoroethylene (PTFE) plate (contact surface diameter of 5 mm) and a highly polished stainless steel spherical surface. Each group of samples in the experiment was diluted to 10 mg mL^−1^ with PBS, and the same volume of PBS was set as a control group for excluding interference. The time‐friction coefficient (COF) curves were recorded during the experiments. To ensure repeatability, the tests were performed in triplicate for each condition.

### Cytotoxicity

*Cell Counting Kit‐8 Method*: Cell Counting Kit (Beyotime, China) was applied to evaluate the cytotoxicity of the material itself on BMSCs. Cells were seeded in 96‐well plates at a density of 0.5 × 10^4^ mL^−1^ and added with MHS (0.1 mg mL^−1^), MHS @ PPKHF (0.1 mg mL^−1^), PPHF (1 µm), and PPKHF (1 µm), respectively. The plates were placed in an incubator at 37 °C in a 5% CO^2^ humidified atmosphere and the medium was changed every 2 days. 90 µL of DMEM medium and 10 µL of CCK‐8 were added to each well at 12, 24, and 48 h and the absorbance was detected at 450 nm using a microplate reader (FlexStation3, Japan) after further incubation for 2 h.

*Live/Dead Staining Experiment*: Cell viability of BMSCs was determined by live/dead staining (Life Technologies). Live cells were stained with calcein AM and dead cells were stained with PI. In short, BMSCs were seeded at a density of 5 × 10^4^ per mL in 24‐well plates and added with MHS (0.1 mg mL^−1^), MHS @ PPKHF (0.1 mg mL^−1^), PPHF (1 µm), and PPKHF (1 µm), respectively. After incubation for 12, 24, and 48 h at 37 °C in 5% CO_2_ atmosphere, the culture medium was aspirated, and the cells were thoroughly washed with PBS before adding the dye solution to each well. After incubation for 40 min at room temperature, the cells were detected by fluorescence microscopy (OLYMPUS, SONY, Japan).

### Immunofluorescence Staining

After 3 weeks of induction, the cell clusters were fixed in 4% paraformaldehyde for 15 min, stained with a rabbit anti‐type II collagen primary antibody (1: 500) (Affinity Biosciences, America) overnight at 4 °C, washed three times with PBS, and incubated for 1 h with FITC‐labeled donkey anti‐rabbit antibody (1:800). After washing three times with PBS, the cytoskeleton and the nuclei were stained with the phalloidin (1: 2000) and DAPI (1: 1000), respectively. Last, the cell clusters were observed under a laser confocal microscope.

### Animal Experiments

All animal procedures were approved by the Animal Ethics Committee of Shanghai Ruijin Hospital, School of Medicine, Shanghai Jiao Tong University (The Ethical Clearance number is SYXK 018–0027).

OA was surgically induced through destabilizing the medial meniscus surgery (Figure S8, Supporting Information). Sprague‐Dawley rats of 12 weeks old (Male, 350–400 g, *n* = 24, Taconic Farms) were anesthetized with isoflurane, subcutaneously injected with meloxicam (1 mg kg^−1^) and buprenorphine sustained release (1 mg kg^−1^), and the right hind limb was skin prepared and disinfected with povidone iodine followed by 70% v/v ethanol solution. The rats were transferred to the operating table, resting on a heated circulating water pad, and covered in a sterile manner. The skin was incised at the right knee from the distal patella to the proximal tibial plateau, and the joint capsule medial to the patellar tendon was further opened with scissors. The patellar tendon was pulled to the lateral side and the fat pad was bluntly separated to expose the medial meniscus tibial ligament (MMTL) of the medial meniscus. So far, the sham operation was completed. The MMTL was opened with microsurgical shears and the anterior foot of the medial meniscus was excised, followed by patella reposition and sequential suturing of the medial joint capsule and skin wound with 5‐0 Polyglecaprone absorbable sutures. After that, the joint was thoroughly rinsed with sterile saline. After surgery, rats were housed individually with free access to food and water and free movement. Meloxicam (1 mg kg^−1^)/q24h and buprenorphine sustained‐release (1 mg kg^−1^)/q48h were injected subcutaneously for 3 days.

The rats were randomized into four groups (six rats in each group). Except for the sham operation group, the other three groups were injected 100 µL of PBS, MHS suspension (10 mg mL^−1^), and MHS@PPKHF suspension (10 mg mL^−1^) into the joint cavity every 3 weeks from the second week after operation. Rats were given running training at 15 m min^−1^ for 1 h on a horizontal treadmill every other day to induce knee OA.

### Visualized Monitoring of Drug Retention in Articular Cavity and Cartilage Repair Process

Twelve extra 12‐week‐old male SD rats (350–400 g) were selected as experimental subjects in the observation group to explore the retention effect and cartilage penetration effect of MHS@PPKHF. The second week after MM*x* surgery, 100 µL of MHS@PPKHF suspension (10 mg mL^−1^) was injected into the joint cavity once. On the day of injection, the fluorescence was detected at the excitation wavelength of 450 nm and emission wavelength of 525 nm by IVIS spectrum system (PerkinElmer, USA) (Figure S9, Supporting Information). 24 h later, three rats were randomly selected and euthanized, and the fluorescence in cartilage tissue was observed by fluorescence microscope (OLYMPUS, SONY, Japan). After that, fluorescence detection and tissue sampling were performed every 2 weeks until the 8th week after surgery, during which the drug was not administered again. All cartilage sections were imaged under the same shooting parameters. The above sections were sagittal sections of the rat knee joint, and those belonging to the femoral medial condyle lesion were selected for observation.

In order to determine the actual drug concentration in cartilage tissue, PPKHF solutions of 1, 2.5, 5, 7.5, and 10 µm were prepared and dropped on the slides, and the fluorescence images were taken by fluorescence microscope (the shooting parameters were consistent with those of tissue sections). Image J software was used to quantify the fluorescence intensity of cartilage tissue sections and different concentrations of PPKHF. Further, the standard curve of fluorescence intensity to drug concentration was created, while the corresponding drug concentrations were obtained according to the values of fluorescence intensity in cartilage tissue.

### X‐Ray and Micro‐CT Evaluation

At 1st week and 8th week after MMx, X‐ray films (exposure time: 5 s, intensity: 6 mAs, voltage: 32 kV) were obtained with an animal X‐ray imager (Faxitron X‐ray, USA) and the width of joint space was measured based on the X‐ray images. Besides, rats were euthanized 8 weeks after surgery and knee specimens were fixed to the slot of a high‐resolution Micro‐CT imaging system (SkyScan1172, Belgium) and scanned at an angle of 0.03°, with a current/voltage of 130 µA per 70 kV, an isometric 20 µm voxel size, and a 100‐ms integration time. 3D rendering was displayed in a consistent density threshold (42.0% of maximum bone mineral density). The measurement of osteophyte volume was similar to previous reports.^[^ ^4^ ^]^ Briefly, osteophytes were identified based on a combination of prominent bone profile and reduced bone mineral density, which included the proximal tibia (4 mm) and distal femur (4 mm). Then, the total osteophyte volume (mm^3^) was calculated for each sample.

### Histological and Immunohistochemical Staining

All rats were euthanized 8 weeks after MM*x*, and the knees were isolated. All samples were fixed (48 hours, 4% paraformaldehyde), decalcified (20 days, 10% EDTA), dehydrated and embedded in paraffin, and finally sectioned at 5 µm. The tissue sections were subjected to HE staining and Safranin O‐fast green staining for histopathological evaluation and the content of glycosaminoglycan in Safranin O‐fast green staining was measured with Image J software. Further, scoring was performed based on the OARSI criteria established by Pritzker et al. in 2006.^[^ ^24^ ^]^

*Immunohistochemical Staining*: A solution of TBS/0.1% Triton X‐100 containing 1% BSA was used for blocking sections after exposure to 3% H_2_O_2_. Then, sections were incubated overnight at 4 °C with a primary antibody against collagen II. The next day, the fluorescence secondary antibody was incubated for 1 h after rinsing the sections. After that, slides were developed with DAB substrate system, scanned with a digital scanner (Carl Zeiss, Germany), and finally measured by Image J software for the collagen II content.

The above sections were sagittal sections of the rat knee joint. The section containing most typical and severe femoral medial condyle lesions was identified for scoring and analysis (Figure 6).

### Statistical Analysis

All experimental data were repeated three times (*n* = 3) and the analysis results were expressed as mean ± SD. One‐way analysis of variance (ANOVA) followed by Turkey test were applied for multiple comparisons. Two‐tailed unpaired *t*‐test was used for comparison between two groups. Differences in significance were indicated as follows: *p < 0.05, **p < 0.01, ***p < 0.001, #*p* < 0.05, ##*p* < 0.01, and ###*p* < 0.001. GraphPad Prism 8.0 (GraphPad Software Inc., USA) was used for statistical analysis.

## Conflict of Interest

The authors declare no conflict of interest.

## Supporting information

Supporting Information

## Data Availability

The data that support the findings of this study are available from the corresponding author upon reasonable request.
