# Supplementary material for: Visualizable and Lubricating Hydrogel Microspheres Via NanoPOSS for Cartilage Regeneration
Source: Adv Sci (Weinh). 2023 Mar 27;10(15):2207438. doi: 10.1002/advs.202207438 (PMC10214257; doi:10.1002/advs.202207438)
Supplement: Supplementary file 1 — Supporting Information [file ADVS-10-2207438-s001.pdf]

Supporting Information

Visualizable and lubricating hydrogel microspheres via nanoPOSS for cartilage regeneration

*Yubin Yao, Gang Wei, Wenguo Cui**

Dr. YB. Yao, G. Wei, Prof. W. Cui
Shanghai Key Laboratory for Prevention and Treatment of Bone and Joint Diseases, Shanghai Institute of Traumatology and Orthopaedics, Ruijin Hospital, Shanghai Jiao Tong University School of Medicine, 197 Ruijin 2nd Road, Shanghai 200025, P. R. China.
E-mail: wgcui80@hotmail.com; wgcui@sjtu.edu.cn.

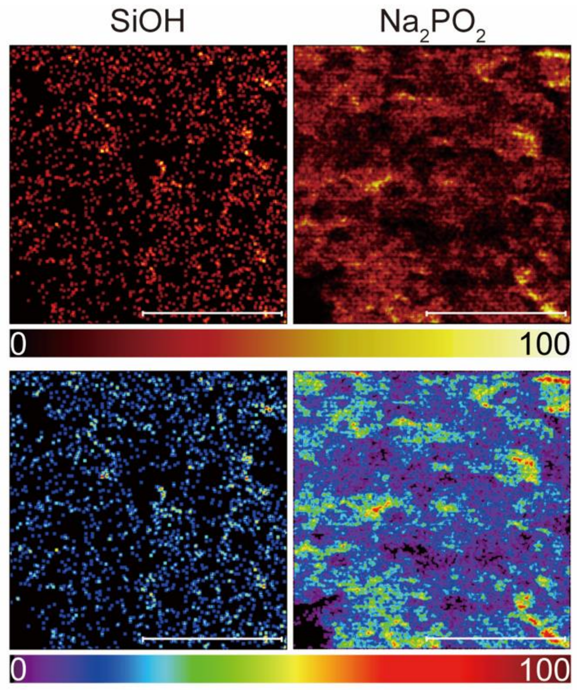

Figure S1: TOF-SIMS Ion Mapping-Positive Ions

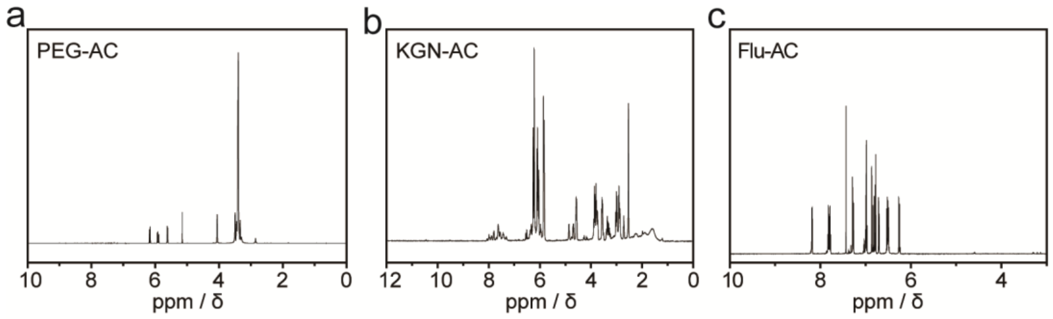

Figure S2: Nuclear magnetic hydrogen (NMH) spectrum of PEG-AC, KGN-AC and Flu-AC.

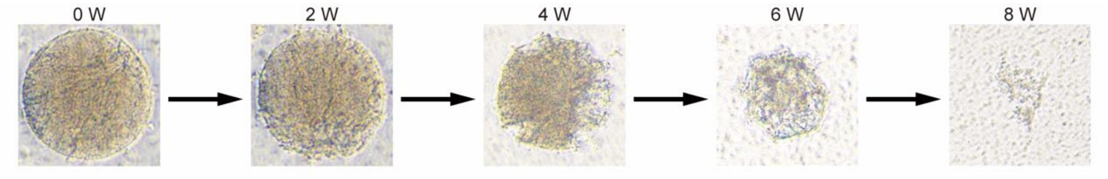

Figure S3**:** Degradation of MHS@PPKHF

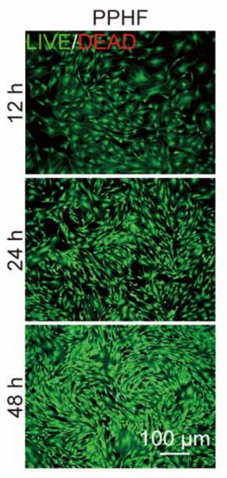

Figure S4: Representative fluorescence images of PPHF co-cultured BMSCs by live-dead staining assay.

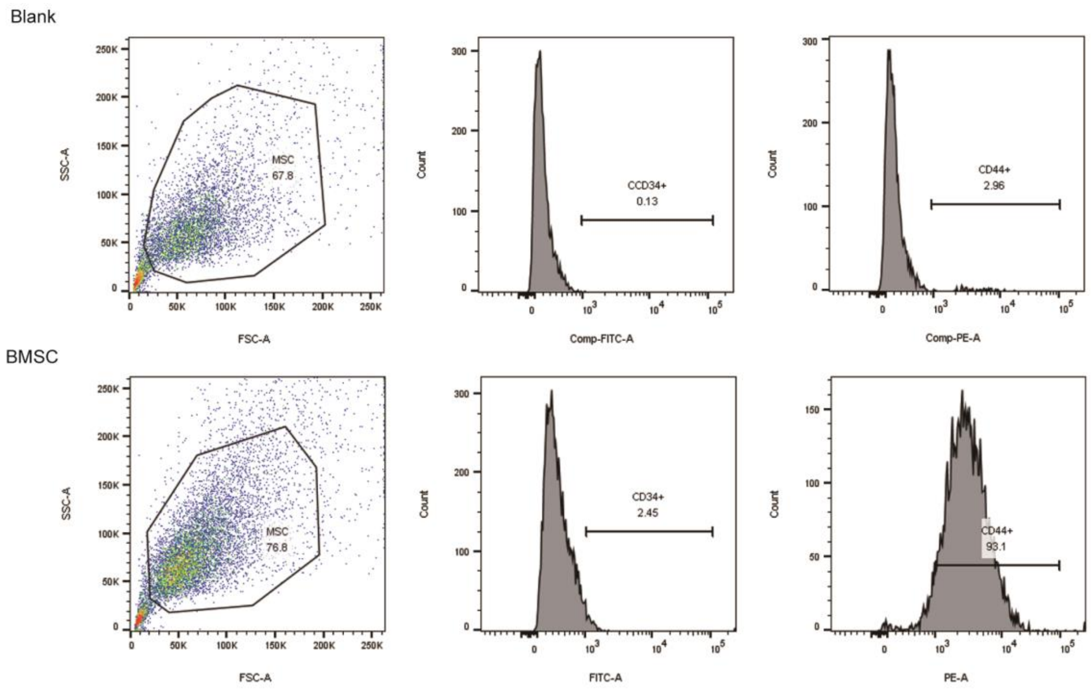

Figure S5: Flow cytometric analysis of BMSCs surface markers

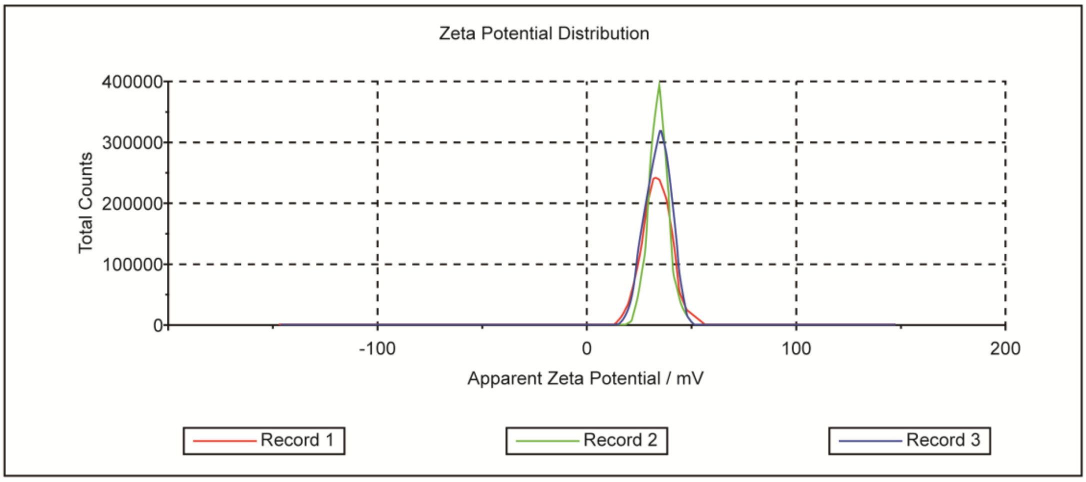

Figure S6: The zeta potential of PPKHF

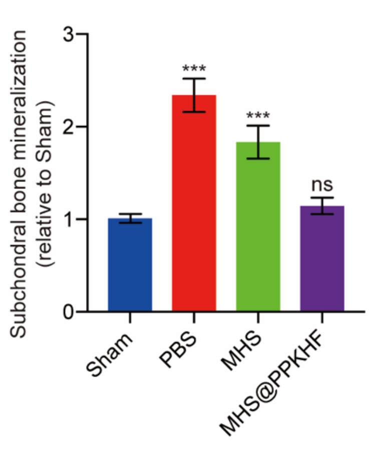

Figure S7: Subchondral bone mineralization (as measured by hydroxyapatite (HA) content, standardized with the sham group)

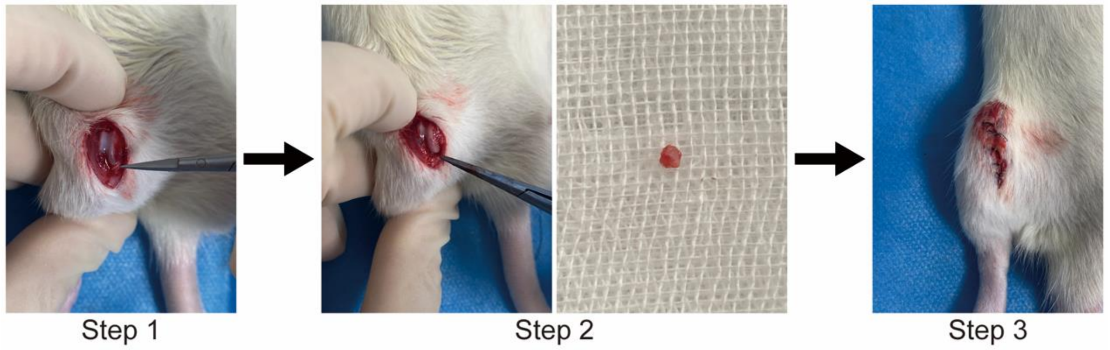

Figure S8: Surgical procedure of establishment rat osteoarthritis model through MMx operation. Step 1. a longitudinal incision of about 1 cm in length from the distal femur to the tibial plateau was made through a medial approach to the knee joint. The skin, muscle and joint capsule were incised layer by layer to expose the medial meniscus. Step 2. Partial resection of the medial meniscus. Step 3. The joint capsule, muscle and skin were sutured layer by layer to close the incision.

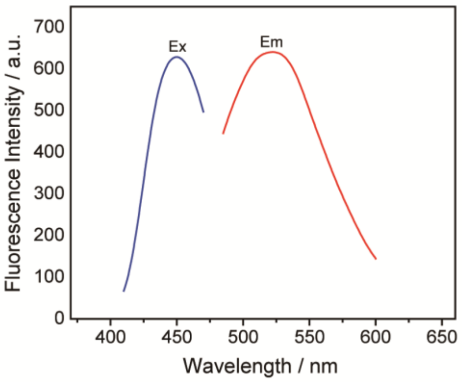

Figure S9: The excitation wavelength and emission wavelength of the fluorescein.

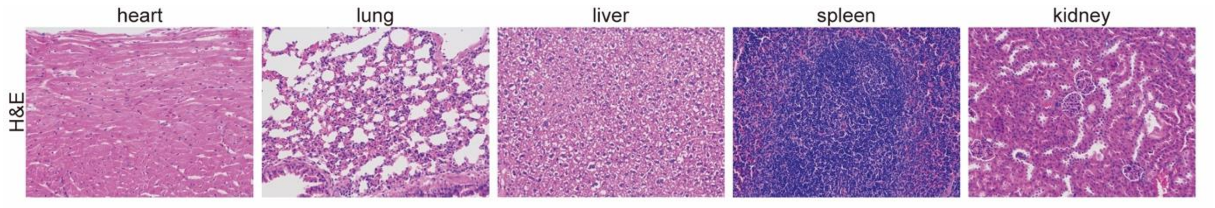

Figure S10: Biosafety assessment of PPKHF: Tissue sections of heart, lung, liver, spleen and kidney from ICR mouse.

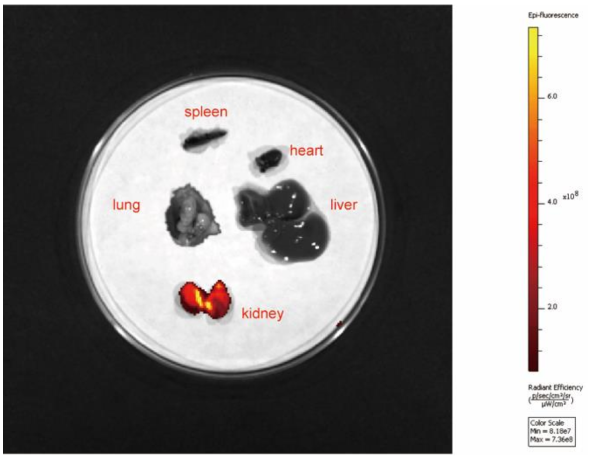

Figure S11: Evaluation of PPKHF metabolism in vivo: Fluorescence imaging of heart, lung, liver, spleen and kidney from ICR mouse.

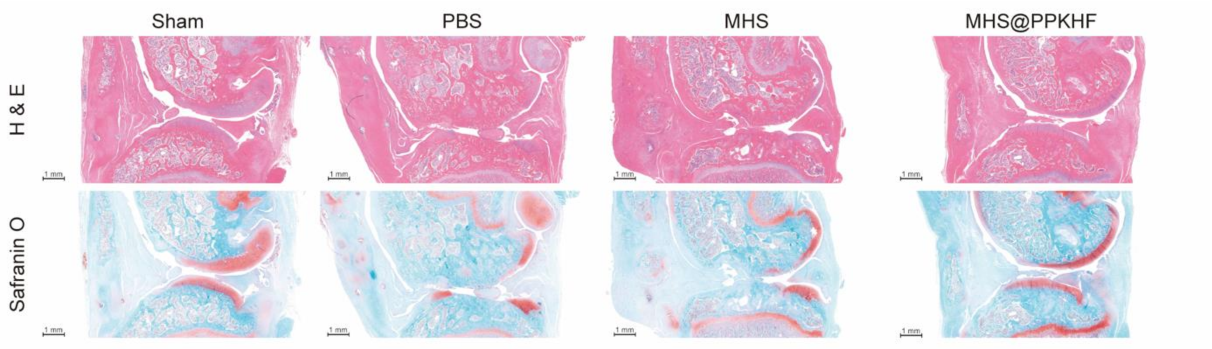

Figure S12: H&E and safranin-o whole sections. Whole sections of H&E and safranin-o staining presented at lower magnification (parent images for the more magnified images provided in Figure 6a).

Table S1. Sequences of primers used for qRT-PCR

Gene	Orientation	Primer sequence
Col II	Forward	ACGCTCAAGTCGCTGAACAACC
	Reverse	ATCCAGTAGTCTCCGCTCTTCC
Aggrecan	Forward	CTGATCCACTGTCCAAGCACCATG
	Reverse	ATCCACGCCAGGCTCCACTC
SOX9	Forward	TCAACGGCTCCAGCAAGAACAAG
	Reverse	CTCCGCCTCCTCCACGAAGG
β-Actin	Forward	CACTATCGGCAATGAGCGGTTCC

	Reverse	CAGCACTGTGTTGGCATAGAGGTC

Table S2. Whole blood test

Species: ICR mouse Model: whole blood

LIST	RESULTS	UNIT	REFERENCE RANGE
WBC	6.2	10^9/L	0.8-6.8
Lymph#	4.8	10^9/L	0.7-5.7
Mon#	0.2	10^9/L	0.0-0.3
Gran#	1.6	10^9/L	0.1-1.8
Lymph%	60.3	%	55.8-90.6
Mon%	2.7	%	1.8-6.0
Gran%	37.0	%	8.6-38.9
RBC	8.24	10^12/L	6.36-9.42
HGB	136	g/L	110-143
HCT	43.5	%	34.6-44.6
MCV	53.6	fL	48.2-58.3
MCH	17.9	pg	15.8-19
MCHC	332	g/L	302-353
RDW	14.8	%	13-17
PLT	503	10^9/L	450-1590
MPV	5.7	fL	3.8-6.0
PDW	17.6		
PCT	0.215	%	

Experimental Section

Fourier transform infrared spectroscopy

The test was performed using a Nicolet 8700 infrared spectrometer (Thermo Fisher, USA). The sample was pressed with KBr powder, and then scanned for 32 times, with scanning wavelength of 4000-500 cm^{-1} and a resolution of 2 cm^{-1}.

Nuclear magnetic resonance (NMR) spectroscopy

Tests were performed with a NMR instrument (Bruker AMX-600, Bruker, Switzerland), with a scanning frequency of 600 MHz. The solvents were D_2O and DMSO-d_6, and tetramethylsilane (TMS) was used as internal standard.

Isolation and culture of Bone marrow mesenchymal stem cells (BMSCs)

BMSCs were obtained from SD rats (male, 4-week-old), and then isolated and cultured in a sterile environment. Bilateral femoral shafts were removed and soaked in sterile PBS containing 500 U/mL penstreptomycin. To expose the marrow cavity, femoral epiphyses were removed, then serum-free DMEM/F12 culture medium was drawn and rinsed with a 5ml syringe. Centrifuge the cell suspension for 5 min at 1000 rpm after pressing through a 200-mesh cell sieve. The cells were suspended in 12% fetal bovine serum and 1% penicillin/streptomycin in DMEM/F12 medium, then incubated at 37 °C under 5% CO_2. BMSCs were isolated from the cell mixture using the differential time adhesion method. The cells were digested with EDTA-trypsin, lysed at 1:3 and re-cultured after reaching about 90% confluence. After three passages of culture and purification, BMSCs in passages 3-6 were used in subsequent experiments.

Flow cytometry

The cells of passage 3 in good growth state were digested with EDTA-trypsin, centrifuged at 4 ℃ and1000 rpm for 5 min, washed for 3 times with PBS containing 1% BSA, and counted. Monoclonal antibodies CD34 and CD44 were added to each tube in turn, while an isotype-

negative control was set up for each sample tube. The resulting cells were incubated on ice in darkness for 45 min, then washed with PBS for three times to remove unbound antibodies and suspended in 500 μL of PBS. Finally, the surface markers of stem cells were identified by flow cytometry.

Induction of chondrogenic differentiation in vitro

(I) Preparation of cell culture medium: Preprepared induction premix (97 mL of basal medium, 1 mL of ITS additive, 10 μL of dexamethasone, 300 μL of ascorbic acid, 100 μL of sodium pyruvate, and 100 μL of proline were mixed well, Cyagen Biosciences Inc, China). 1 mg MHS @ PPKHF was previously soaked in 5 mL of the above premixed solution for 3 weeks, after which 1 mL of supernatant was collected every 3 days for cell culture while the same volume of premix solution was supplemented.

(II) Induction of chondrogenic differentiation of BMSCs: The BMSCs of passage 3 were selected for induction experiment. Cells were digested and counted before differentiation induction. A total of 4×10^5 cells were transferred to a 15 mL centrifuge tube, centrifuged at 250 g for 4 min and re-suspended with the premixed solution, and centrifuged at 150g for 5min twice to wash the cells. After that, 1 mL of the cell culture medium was added and centrifuged again at 150 g for 5 min before incubated at 37 °C in 5% CO_2 atmosphere. Thereafter, the culture medium was changed every 3 days. In the control group, the culture medium was composed of 1 mL premix and 10 μL TGF-β, and then processed the same as test group.

(III) qRT-PCT: Total RNA was extracted from the cell clusters after 3 weeks of induction and reverse transcribed to cDNA using the PrimeScript TM RT kit (TaKaRa, Japan) according to the manufacturer's instructions. A 20-μL reaction system including 2 μL of cDNA was then subjected to RT-PCR on an Applied Biosystems 7500 Fast Real-Time RCR System (Applied Biosystems, Foster City, USA) using the SYBR PrimeScript RT-PCR kit (TaKaRa, Japan) (primer sequences were shown in Table S1). Finally, the relative expression of the indicated

genes (Aggrecan, Col2 and SOX-9) was calculated by the 2 ^ -ddCt method, and the expression of all genes was standardized to the TGF-β treated group. The experiment was performed in triplicate.

Biosafety assessment

(I) Potentially inflammatory effects of the system: Three ICR mice were intraperitoneally injected with 1ml PPKHF nanoparticles at a concentration of 1 μM. After 4 weeks, the mice were euthanized, and the whole blood was taken for testing. Meanwhile, major organs (heart, lung, liver, spleen and kidney) were taken for HE staining for pathological analysis.

(II) Metabolic assessment: Major organs (heart, lung, liver, spleen and kidney) of ICR mice were taken for fluorescence imaging.
